# Wanted—A Bureau of Facts

**Published:** 1914-03

**Authors:** 


### Wanted—A Bureau of Facts

Every physician's attention is called, from time to time, to the need of being able to secure, readily, information as to what is already known. A contributor, after spending much time and trouble in solving a difficult problem, published an article announcing his achievement and, while his article was being printed, wrote in haste to give credit to the prior accomplishment of the same thing in the same way. Another man published an article of comparative novelty, almost duplicating one on the same subject, published in the same journal within two years, the case being especially embarrassing because the earlier author had given credit to the later one for certain preliminary work, whereas the later one ignored the former. The very grossness of the plagiarism was the best evidence that it was entirely innocent. One of the best talking points of anti-vivisectionists is that experiments are unnecessarily duplicated. Research work of all kinds, involving expense, not merely of money but of the labor of brilliant men whose lives are all too short, is repeated several times over, because of the inaccessibility of the data of other workers. A phenomenon, rare and suggestive of the most valuable scientific results or involving tremendous humanitarian advance is passed unnoticed, simply because the observer—or rather one who might have been truly an observer—had not had his attention called to the possible existence of a condition well known to someone else. Misleading and one-sided deductions are drawn by a dozen men, each from a single experience, whereas if the whole dozen experiences could be collated, not necessarily in time, the true significance and the existence of a general principle might have been appreciated.

We have been impressed, time and again, with the difficulty of obtaining definite information on points suggested in practice, beyond the stock in trade of the ordinary text book writer, even when it would seem that the point in question was of the simplest kind. For example, it was several years after gastric analyses were fluently talked about before one could obtain definite statements of normal standards and the real significance of a given variation from the standards. We questioned for several years before we could learn whether the little bright red arterio-capillary angiomata, so commonly observed in the skin, were or were not congenital. We are not absolutely positive yet on this point, but we think that statements by physicians and patients that such spots have appeared where they did not originally exist, are more reliable than the first off-hand assurances that they are congenital. Gonorrhœa is a common disease, so common that, by the law of chance, it must not very rarely exist in a patient who is infected with another disease of febrile nature. Yet it took considerable effort to learn what effect the second infection had on the first. Some time ago the question was raised as to whether or not in hot climates, where the atmospheric temperature was higher than that of the body, any allowance or special precautions had to be taken in clinical thermometry. How many can answer the question off-hand?

It may be said that the various cyclopædias and indexes of medical literature ought to furnish needed information on any point. But, in our experience, such a search is about like trying to find an unknown foreign word in a dictionary printed only in the same foreign language. If one knows the word or the fact, he does not need to look it up, except for certain details; if he does not know it, he does not know where to look. Even in simple matters of history it is often difficult to obtain information. For example, a few years ago, in seeking light on a case of chylous mesenteric cyst, we found every reference dating back to Rokitanski's case in 1842. Later, we found a perfectly plain case recorded in this country several years earlier and others, in foreign volumes, antedating Rokitanski's case by almost 150 years.

The only method that we can suggest for properly compiling knowledge is the establishment of living, human bureaus of information, properly correlated and rendered available by some form of index. But the details are not so easy to work out. Large endowment, wide scope, probably governmental support and international co-operation seem to be required. And there is one more factor to be considered—broad minded, generous co-operation. No mere clerical and routine indexing will solve the problem. A classic, though extreme illustration of the failure of this method is the work of a conscientious but mechanical compiler who prepared an index with the following items:

Lead, kindly light
" , poisoning.

---

This issue begins with page 471; the corresponding issue of 1913 began with page 437; that of 1912 with page 421; that of 1911 with page 407. In other words, with the close of the seventh issue of the current volume, the equivalent of an extra full monthly issue has been supplied to our readers. That this rate of progress may be continued, we ask co-operation in the way of securing additional subscriptions and advertisements, and especially mention of our publicity in writing to manufacturers.

